# Potential of Visible and Near-Infrared Hyperspectral Imaging for Detection of *Diaphania pyloalis* Larvae and Damage on Mulberry Leaves

**DOI:** 10.3390/s18072077

**Published:** 2018-06-28

**Authors:** Lingxia Huang, Liang Yang, Liuwei Meng, Jingyu Wang, Shaojia Li, Xiaping Fu, Xiaoqiang Du, Di Wu

**Affiliations:** 1 College of Animal Sciences, Zhejiang University, Hangzhou 310058, China; lxhuang@zju.edu.cn (L.H.); lyoung1101@163.com (L.Y.); 21517069@zju.edu.cn (L.M.); m17794539235@163.com (J.W.); 2 College of Agriculture & Biotechnology, Zhejiang University, Zijingang Campus, Hangzhou 310058, China; 11216044@zju.edu.cn; 3 Zhejiang Provincial Key Laboratory of Horticultural Plant Integrative Biology, Hangzhou 310058, China; 4 The State Agriculture Ministry Laboratory of Horticultural Plant Growth, Development and Quality Improvement, Hangzhou 310058, China; 5 Faculty of Mechanical Engineering & Automation, Zhejiang Sci-Tech University, Hangzhou 310018, China; fuxp@zstu.edu.cn (X.F.); xqiangdu@zstu.edu.cn (X.D.); 6 Key Laboratory of Transplanting Equipment and Technology of Zhejiang Province, Hangzhou, 310018, China; 7 South Taihu Agricultural Technology Extension Center in Huzhou, Zhejiang University, Huzhou 313000, China

**Keywords:** hyperspectral imaging, mulberry leaves, *Diaphania pyloalis*, larvae, damage

## Abstract

Mulberry trees are an important crop for sericulture. Pests can affect the yield and quality of mulberry leaves. This study aims to develop a hyperspectral imaging system in visible and near-infrared (NIR) region (400–1700 nm) for the rapid identification of *Diaphania pyloalis* larvae and its damage. The extracted spectra of five region of interests (ROI), namely leaf vein, healthy mesophyll, slight damage, serious damage, and *Diaphania pyloalis* larva at 400–1000 nm (visible range) and 900–1700 nm (NIR range), were used to establish a partial least squares discriminant analysis (PLS-DA) and least-squares support vector machines (LS-SVM) models. Successive projections algorithm (SPA), uninformation variable elimination (UVE), UVE-SPA, and competitive adaptive reweighted sampling were used for variable selection. The best models in distinguishing between leaf vein, healthy mesophyll, slight damage and serious damage, leaf vein, healthy mesophyll, and larva, slight damage, serious damage, and larva were all the SPA-LS-SVM models, based on the NIR range data, and their correct rate of prediction (CRP) were all 100.00%. The best model for the identification of all five ROIs was the UVE-SPA-LS-SVM model, based on visible range data, which had the CRP value of 97.30%. In summary, visible and near infrared hyperspectral imaging could distinguish *Diaphania pyloalis* larvae and their damage from leaf vein and healthy mesophyll in a rapid and non-destructive way.

## 1. Introduction

China’s sericulture production has a history of several thousand years [1] and has formed a complete industry with certain economic value. In 2017, the domestic sales volume of silk goods in China was 720 million U.S. dollars, which is an increase of 7.1% over the previous year. The exports amounted value of silk goods was 3.56 billion U.S. dollars, a year-on-year increase of 22.9% [2]. Sericulture in China shows a good development prospect. As one of the most essential production materials of sericulture, mulberry leaves are the only feed source for silkworms. The yield and quality of mulberry leaves directly restrict the development of sericulture. In 2017, the total area of mulberry fields in China reached 788.73 thousand hm^2^, which is an increase of 24.7%, compared with 632.5 thousand hm^2^ in 2000 [2]. In the past ten years, despite the increase in the planting area of mulberry trees, the production of mulberry leaves suffered a great loss due to insect pests.

*Diaphania pyloalis* Walker (Lepidoptera: Pyralididae), which is also called *tortrix moth*, is a monoxenous parasite and one of the most common mulberry pests [3]. *Diaphania pyloalis* nibbles the leaves and poses a great threat to the production of mulberry leaves. The excretion of *Diaphania pyloalis* also can seriously affect the quality of mulberry leaves and pose a serious threat to the production of cocoon and silkworm eggs [4]. Currently, *Diaphania pyloalis* occurs in not only the main sericulture areas of China, such as the Zhejiang, Jiangsu, Guangxi, Guangdong, Sichuan, and Hubei provinces, but also other countries and regions including Japan, India, and Southeast Asia. The control of mulberry pests is the focus of the current management in mulberry fields. The sericulture industry needs to develop a rapid and nondestructive detection technique for *Diaphania pyloalis*, thereby providing silkworms with high-quality mulberry leaves as feed.

Visual inspection is a common method of pest detection. Nevertheless, manual observation is laborious, costly, and tedious. Moreover, because larvae are small and their color is very similar to that of the mulberry leaves, it is error-prone in the pest detection based on naked eyes. On the other hand, a computer vision technique can measure the objective results for identifying pests and their damage in a low cost way [5,6]. Different from visual inspection, image detection is not interfered by human factors, so it can reduce the interference that is caused by subjective factors. Nevertheless, computer vision technology cannot distinguish samples with similar colors, because it mainly detects monochrome or color images in the visible spectral range [7]. Because of the similar color of *Diaphania pyloalis* and mulberry leaves, it is difficult to distinguish between pests and leaves by visual observation or computer vision technique.

As an extension of computer vision technique, hyperspectral imaging (HSI) is widely used for the quality inspection of a variety of food and agricultural products [8,9,10], and also plant diseases [11,12,13] and insect pests [14,15,16]. HSI can simultaneously obtain the spectral and spatial information of the sample and form a three-dimensional (3-D) data cube. One dimension in the three-dimensional ‘hypercube’ is the spectral wavelength, and the other two dimensions are the spatial coordinates (x, y) of the pixels in the hyperspectral image [17]. Therefore, HSI can use the spectral information to distinguish between pests and leaves, and to locate the spatial position of pests through the spatial information contained in the cube. Nevertheless, so far, little research has been reported on the rapid detection of *Diaphania pyloalis* and their damage on mulberry leaves, based on HSI technology.

In this work, visible and near-infrared (Vis-NIR) hyperspectral reflectance imaging techniques were used to distinguish the *Diaphania pyloalis* larva and leaves, and to identify the pest damage in a rapid and objective way. The successful outcome of the work is very beneficial for providing silkworm farmers with high-quality mulberry leaves, thereby increasing the yield of silkworms and improving the quality of silk. The specific research content includes the following aspects. (1) The measurement of hyperspectral images in the spectral range of visible and near-infrared (400–1700 nm). (2) The extraction of the characteristic spectra of *Diaphania pyloalis* and mulberry leaves. (3) Establishment of classification models between leaves and damage, leaves and *Diaphania pyloalis* larvae, damage and *Diaphania pyloalis* larvae, and all samples by multivariate analysis. (4) The identification of a few important wavelengths that are most useful for the classification. (5) The comparison of two hyperspectral imaging systems working in the ranges of 400–1000 nm and 900–1700 nm, respectively.

## 2. Materials and Methods

### 2.1. Sample Preparation

The mulberry leaves (Dashi) were picked up from a local mulberry field in Zijingang campus, Zhejiang University, Hangzhou, China, 30°18′20′′ N, 120°5′53′′ E on 24 October 2017. Then, leaves with no defects other than larva damage were selected. There were three kinds of leaves obtained (120 leaves), namely healthy leaves (35 leaves), leaves with larva damage (70 leaves), and leaves with larvae (15 leaves).

### 2.2. Hyperspectral Imaging System and Data Acquisition

The hyperspectral imaging system consisted of two parts. One was the spectral acquisition unit, which was in a large black box. The spectral acquisition unit had two cameras, two imaging spectrographs, and two adjustable 150 w quartz tungsten halogen lamps. The two spectrographs measured the hyperspectral images in 400–1000 nm (visible range) and 900–1700 nm (NIR range), respectively. The black box was closed during data collection to avoid the influence of external light. The other part was the control unit, which had a conveyer belt, a stepper motor, and a computer.

During data acquisition, mulberry leaves were placed on the conveyer belt and moved under lens line by line. Both the visible range data (400–1000 nm) and NIR range data (900–1700 nm) of each leaf were obtained. Visible range data was measured by the spectrograph with a Si detector (ImSpectorV10E, Spectral Imaging Ltd., Oulu, Finland). The NIR range data was measured by the spectrograph with an InGaAs detector (ImSpectorN17E, Spectral Imaging Ltd., Oulu, Finland). The hyperspectral images of all 120 leaves were measured.

### 2.3. Correction of Hyperspectral Images

After data collection was completed, and the 3-D data cubes containing both spectral (λ) and spatial (x, y) information were obtained. The raw image signal collected was the signal intensity, not the reflection spectrum of the sample. Therefore, the raw images were corrected into hyperspectral images using the following equation based on white and dark image references.

(1) $$R=\frac{I_{R}-I_{D}}{I_{W}-I_{D}}\times 100.$$

where *I_R_* is the raw hyperspectral image, *I_D_* is the dark image with a reflectance closed to 0, *I_W_* is the white image, which was measured based on a Teflon white board and had a reflectance close to 99%. To capture the dark image, the light was turned off and the lens was covered by an opaque cap. The dark reference could also eliminate the dark current in the raw data. The data acquisition process for both the dark image and white image was consistent with the image acquisition process of samples. The corrected images were used for subsequent calculations, including spectral extraction, variable selection, and model establishment.

### 2.4. Data Analysis

A function called the region of interests (ROI) in ENVI v4.6 software (Research Systems Inc., Boulder, CO, USA) was used to manually identify the ROIs of the samples. Based on the corrected images, the ROIs of leaf vein, healthy mesophyll, slight damage, serious damage, and larva were obtained. There were five classes of ROIs (leaf vein, healthy mesophyll, slight damage, serious damage, and larva) obtained in this work. Each ROI was a sample. As a result, there were 40, 34, 32, 28, and 12 ROIs/samples obtained from the visible hyperspectral images (400–1000 nm) for the leaf vein, healthy mesophyll, slight damage, serious damage, and larva, respectively. From the NIR hyperspectral images (900–1700 nm), there were 40, 34, 33, 34, and 15 ROIs/samples obtained for the leaf vein, healthy mesophyll, slight damage, serious damage, and larva, respectively. The numbers of pixels in the ROIs in the visible hyperspectral images were generally between 100 and 400 for leaf vein, 4000 and 12,000 for healthy mesophyll, 400 and 2000 for slight damage, 1200 and 4800 for serious damage, and 200 and 800 for larva. On the other hand, the numbers of pixels in ROIs in NIR hyperspectral images were generally between 25 and 100 for leaf vein, 1000 and 3000 for healthy mesophyll, 100 and 500 for slight damage, 300 and 1200 for serious damage, and 50 and 200 for larva. The mean value of all pixels in one ROI was calculated to obtain the representative spectrum of this ROI (sample). The representative spectrum of each sample was combined into a spectral matrix (*X*). Each column of the matrix was a variable and represented one wavelength. There were 428 variables for visible range data and 256 variables for NIR range data. Each row of the matrix represented a sample. On the other hand, there were five classes of ROIs, which were leaf vein, healthy mesophyll, slight damage, serious damage, and *Diaphania pyloalis* larva. For the classification, natural numbers 1, 2, 3, 4, and 5 were used to represent the above five classes. The class numbers of all samples were combined into a column vector (*Y*).

Since there were hundreds of variables for visible range data and NIR range data, a multivariate analysis was used to establish a quantitative relationship model between matrix *X* and vector *Y*. Matrix *X* was the independent variables and vector *Y* was a dependent variable. Two supervised multivariate classification methods of the partial least squares discriminant analysis (PLS-DA) [18,19] and least-squares support vector machines (LS-SVM) [20,21] were used for model calibration. PLS-DA extracts the latent variables (LVs) by decomposing independent variables and the dependent variable simultaneously, and establishes the regression model based on a few informative LVs. The LS-SVM maps input variables to a high-dimensional space through a nonlinear mapping function for classification and regression analysis. Therefore, there were two types of models that were established based on full variables, namely the PLS-DA model established based on full variables (F-PLS-DA model) and the LS-SVM model established based on full variables (F-LS-SVM model). It should be noted that for establishing every classification model, all samples were randomly divided into a set of calibration (three quarters of the samples) and another independent set for prediction (one quarter of the samples). Moreover, a large number of spectral variables usually have variables with low signal-to-noise ratios or useless for the models establishment. Removing these redundant variables proves to improve the model accuracy and robustness in some applications. Successive projections algorithm (SPA) [22,23], uninformation variable elimination (UVE) [24,25], UVE-SPA [26,27], and competitive adaptive reweighted sampling (CARS) [28,29] methods, which are commonly used in the selection of near-infrared spectroscopy variables, were used to select wavelengths that could discriminate insects and damage from the leaves. Also, there were eight types of models based on the selected variables, which are the PLS-DA model based on variables selected by SPA (SPA-PLS-DA model); the LS-SVM model based on variables selected by SPA (SPA-LS-SVM model); the PLS-DA model based on variables selected by UVE (UVE-PLS-DA model); the LS-SVM model based on variables selected by UVE (UVE-LS-SVM model); the PLS-DA model based on variables selected by UVE-SPA (UVE-SPA-PLS-DA model); the LS-SVM model based on variables selected by UVE-SPA (UVE-SPA-LS-SVM model); the PLS-DA model based on variables selected by CARS (CARS-PLS-DA model); and the LS-SVM model based on variables selected by CARS (CARS-LS-SVM model). The performance of the classification models was evaluated with the correct rate. The correct rate was calculated by dividing the number of samples correctly classified by the number of all of the samples. When the absolute value of the difference between the known and predicted class number of a sample was less than or equal to 0.5, the sample was considered to be correct. Otherwise, this sample was considered misjudged. There were three correct rate indicators to evaluate the performance of the model. They were the correct rate of calibration (CRC), correct rate of prediction (CRP), and the absolute difference between the correct rate of calibration and prediction (AB_CR). AB_CR was used to evaluate the robustness of the model. The smaller the AB_CR, the better the model’s robustness. All calculations of model establishment, variable selection, and model evaluation were done in Matlab R2015b software (MathWorks Inc., Natick, MA, USA).

## 3. Results

### 3.1. Spectral Features and Images of Leaves, Larvae, and Damage

**Figure 1:**
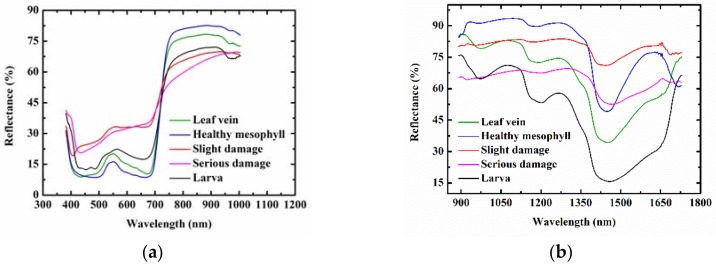

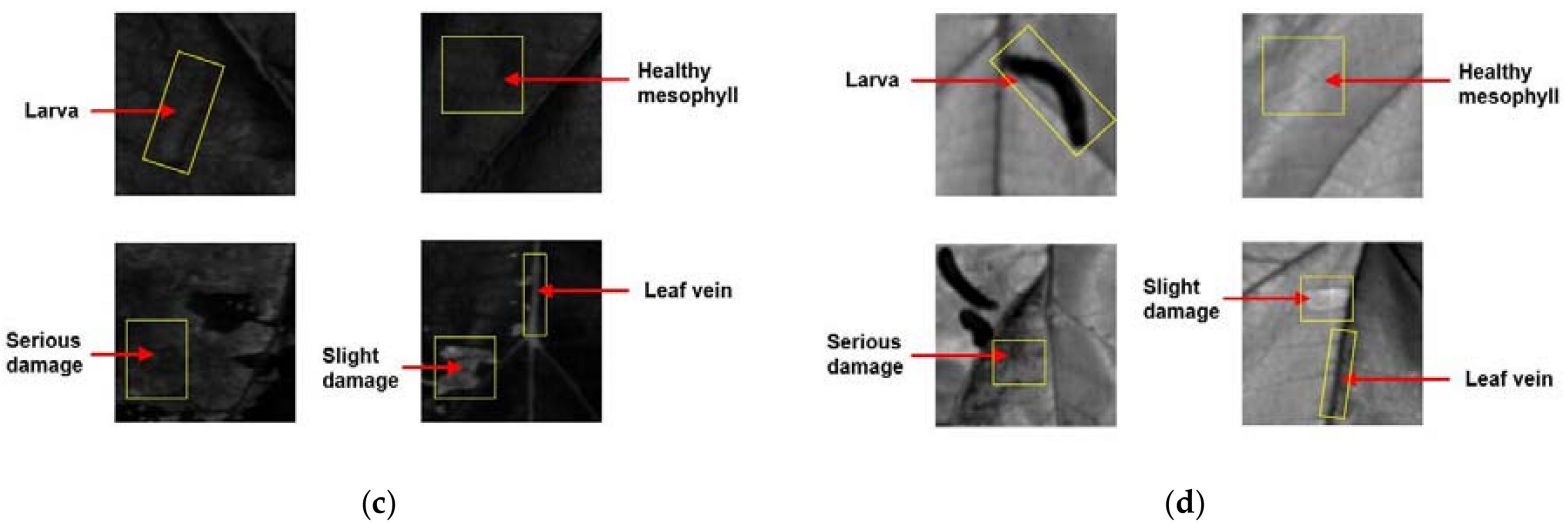
Extracted mean representative spectra and images of leaf vein, healthy mesophyll, slight damage, serious damage, and *Diaphania pyloalis* larva: (**a**) 400–1000 nm (visible range) and (**b**) 900–1700 nm (near-infrared [NIR] range); (**c**) 660 nm and (**d**) 1250 nm.

Figure 1 shows the representative spectra of leaf vein, healthy mesophyll, slight damage, serious damage, and *Diaphania pyloalis* larva in the wavelength ranges of 400–1000 nm (visible range) and 900–1700 nm (NIR range). The visible spectral bands between 400–700 nm in the visible range data mainly reflected the color information of different ROI classes. The representative spectra of leaf vein, healthy mesophyll, and *Diaphania pyloalis* larva had similar patterns in the visible spectral bands, which showed why the *Diaphania pyloalis* larvae on mulberry leaves were difficult to be identified by naked eyes. Green bands had a higher reflectance than the red and blue bands for the representative spectra of leaf vein, healthy mesophyll, and *Diaphania pyloalis* larva, which explained why they were green. The representative spectra of slight damage and serious damage had higher reflectance than those of the leaves and larvae, especially in the red bands. In the NIR spectral bands, different chemical molecules have their own absorption peaks, which is very important for predicting sample quality and differentiating between different sample categories. Nevertheless, these absorption peaks mainly overlap in several NIR bands, resulting in no obvious spectral absorption peaks in the NIR band, but only some broadband peaks. A main absorption band in the NIR range was observed at around 1440 nm, which was assigned to O–H, the first stretching overtone of water [30]. In general, this peak was relatively flat for damage, while it had a strong spectral absorption for leaves and larvae. Another main absorption for NIR range data was at around 1200 nm, which was assigned to the C–H stretching second overtone [31]. In addition, there was another water absorption was at around 970 nm, which could be found in both the visible range data and NIR range data and was assigned to the O–H second stretching overtone of water [32]. Only the larvae had a strong absorption of this peak, as shown in both Figure 1a,b. This peak of the leaf vein, healthy mesophyll had a weak absorption, and this absorption peak of the insect pests could hardly be observed. In the short-wave NIR region (700–1000 nm) in the visible range data, the spectral curves of the leaf veins and mesophyll were similar, and the spectral curve of serious damage was different from other ROI classes. For the NIR range data, the representative spectra varied among the different ROI classes. However, Figure 1 shows the average representative spectra of different kinds of ROI classes. When the spectrum of each ROI sample was displayed in one figure, there was a serious overlap between each other, making it impossible to identify the different ROI classes directly by analyzing the spectrum. In addition, examples of the hyperspectral images of the leaf vein, healthy mesophyll, slight damage, serious damage, and *Diaphania pyloalis* larva in the visible range and NIR range have been supplemented in Figure 1. Because there were 428 and 256 images at different spectra wavelengths in the visible range and NIR range, respectively, only the images at 660 nm and 1250 nm were selected as examples, shown in Figure 1. It can be seen that the images at only one wavelength could not distinguish between the five classes of samples. The images at several important wavelengths should be considered. Therefore, a multivariate analysis was used to mine the characteristic spectral information at some important wavelengths to establish classification models for distinguishing the different ROI classes.

### 3.2. Classification of Leaf Vein, Healthy Mesophyll, Slight Damage, and Serious Damage

The classification models for identifying the four classes of samples (leaf vein, healthy mesophyll, slight damage, and serious damage) were established by PLS-DA, LS-SVM, and linear discriminant analysis (LDA) algorithms, respectively. The matrixes of both the visible range data and the NIR range data were used to establish the models, the results are shown in Table 1.

Firstly, full variables were used for the model establishment. When the visible range data was considered, the PLS-DA model with full variables (F-PLS-DA) had the CRC value of 100% and the CRP value of 82.35%. The LS-SVM model with full variables (F-LS-SVM) had a better performance than the F-PLS-DA model. The CRP of the F-LS-SVM model was 97.06%, which was 14.71% higher than that of the F-PLS-DA model. Moreover, the AR_CR of the F-LS-SVM model was only 2.94%, whereas that of the F-PLS-DA model was 17.65%. Therefore, the F-LS-SVM model was more robust than the F-PLS-DA model. And then, on the basis of the full variables, the variable selection calculations were carried out. When the SPA was calculated, only 10 important variables were selected as they had the lowest root mean square error of the multiple linear regression (MLR) models. The MLR models were established based on the candidate subsets of the variables obtained by a sequence of projection in the SPA calculation. PLS-DA and LS-SVM were then used to establish the classification models based on the selected variables. It was found that the SPA-PLS-DA model had a similar prediction to the F-PLS-DA, and the prediction accuracy of the SPA-PLS-DA model was also similar to that of the F-PLS-DA model. Therefore, it shows that the ten variables obtained through variable selection could represent all 428 variables, so as to identify the leaves and damage. A UVE calculation was also carried out, and the most informative 266 variables were selected. The UVE-PLS-DA model had a similar prediction to the F-PLS-DA model. The UVE-LS-SVM model obtained 100% correct rates for both the calibration and prediction sets. Further SPA calculation was performed on the basis of the UVE variable selection results, and the PLS-DA and LS-SVM models were established based on the obtained variables. The results showed that, compared with UVE-based models, the UVE-SPA calculations could not further improve the model accuracy, but could significantly reduce the number of variables (266 vs. 9). At last, CARS was used for the variable selection. There were 28 variables selected by CARS, and the prediction accuracy of the established CARS-PLS-DA and CARS-LS-SVM models was similar to that of the full-variable models. Through an overall analysis of the above results, it could be seen that a small number of variables that were most useful for the classification between leaves and damage could be selected through the variable selection.

When the NIR range data was considered, in general, the results of the models that were established based on the NIR range data were better than those based on the visible range data, especially for the PLS-DA models. The CRP of the F-PLS-DA model based on the NIR range data was 97.22%, whereas the CRP of the F-PLS-DA model based on visible range data was 82.35%, which had declined by 14.87%. A similar phenomenon existed for the result of the variable selection. The average CRP of four PLS-DA models after the variable selection (SPA, UVE, UVE-SPA, and CARS) based on the NIR range data was 95.83%, whereas that based on the visible range data was only 79.41%. The best model was the SPA-LS-SVM model based on NIR range data, which had a CRP value of 100% and only nine input variables. The above results showed that the hyperspectral imaging in both the visible and short-wave NIR region and long-wave NIR region could be used to distinguish leaf vein, healthy mesophyll, slight damage, and serious damage.

### 3.3. Identification of the Diaphania pyloalis Larvae

The number of pests on the leaves can be used to forecast the pest severity in the future. Therefore, it was important to identify the *Diaphania pyloalis* larvae from the background of the mulberry leaves. The classification models for identifying three classes of samples (leaf vein, healthy mesophyll, and *Diaphania pyloalis* larva) were established by PLS-DA, LS-SVM, and LDA algorithms, respectively. The matrixes of both the visible range data and NIR range data were used to establish the models, and the results are shown in Table 2.

When the full variables were used for the model calibration, the performances of the F-PLS-DA model and the F-LS-SVM model, based on visible range data, were similar. The average CRP of these two models was 93.18%. On the other hand, the performances of the F-PLS-DA model and the F-LS-SVM model, based on NIR range data, were different. The CRP of the F-LS-SVM model was 100%, whereas that of the F-PLS-DA model was only 60.87%. Moreover, compared with the F-LS-SVM model, whose AB_CR was 0%, the AB_CR of the F-PLS-DA model was 31.55%. That means the F-LS-SVM model was much more robust than the F-PLS-DA model. For the NIR range data, the PLS-DA algorithm also had a much poorer prediction than the LS-SVM algorithm after the variable selection. The average CRP of the LS-SVM models based on four variable selection algorithms was 100%, whereas that of the PLS-DA models after the variable selection was only 61.96%. Different from analyzing the NIR range data, the PLS-DA algorithm obtained a good prediction when analyzing the visible range data. The average CRP of the PLS-DA models based on four variable selection algorithms was 82.95%. Nevertheless, the results of the LS-SVM models after the variable selection were still better than those of the PLS-DA models. The average CRP of the LS-SVM models after the variable selection was 95.45%. There were five models that obtained a 100% CRP value. They were the F-LS-SVM, SPA-LS-SVM, UVE-LS-SVM, UVE-SPA-LS-SVM, and CARS-LS-SVM models based on the NIR range data. The numbers of the variables selected by SPA, UVE, UVE-SPA, and CARS were reduced from 256 to 9, 156, 15, and 13, respectively. The best model for identification between leaves and larvae was the SPA-LS-SVM model, based on the NIR range data, because it had only nine input variables, which was the lowest, compared with the other models with a 100% CRP value. While ensuring the model prediction accuracy, the number of input variables of the SPA-LS-SVM model decreased by 96.48% (256 vs. 9), compared with that of the F-LS-SVM model. The above results showed that leaf vein, healthy mesophyll, and *Diaphania pyloalis* larva could be distinguished using hyperspectral imaging in both the visible as well as the short-wave region and long-wave NIR region.

### 3.4. Classification of Diaphania pyloalis Larvae and Their Damage

The damage caused by larvae can be used to evaluate the current severity of the damage, which is important to determine the quality of SPA—successive projections algorithm; PLS-DA—partial least squares discriminant analysis the mulberry leaves. However, without larvae, such damage will not continue to increase. On the other hand, if there are larvae on the leaves, the damage will increase and cause greater losses. In other words, the number of larvae is related to the severity of the pest and losses of the mulberry leaves in the future. Therefore, the larvae and their damage are two different factors to evaluate the severity of a larva infection, and it is important to carry out the classification between the larvae and their damage. The classification models for identifying three classes of samples (slight damage, serious damage, and *Diaphania pyloalis* larva) were established by PLS-DA, LS-SVM, and LDA algorithms, respectively. The matrixes of both the visible range data and NIR range data were used to establish the models, and the results are shown in Table 3.

It could be seen from the results of the visible range data, that the CRP value of both the F-PLS-DA model and F-LS-SVM model were 100%. Variable selection calculations were carried out. The UVE achieved a good result with the CRP value of 100%. Nevertheless, there were still 249 variables after the UVE calculation. SPA was further carried out based on the variables selected by the UVE algorithm. Although the number of variables that were selected by the UVE-SPA algorithm decreased significantly, the UVE-SPA model did not achieve a 100% CRP value. For the calculation of CARS and SPA, both the PLS-DA algorithm and the LS-SVM algorithm obtained 100% CRC and CRP, and the numbers of the input variables were only 13 and 20, respectively.

When the NIR range data was considered, the CRP value of the models all exceeded 90%, regardless of whether they were based on full variables or selected variables. The CRP value of the F-LS-SVM model was 100%, which was better than that of the F-PLS-DA model, which had the CRP value of 95.24%. Compared with the models that were established before the selection of the variables, the accuracy of the models, established based on the selected variables, was not improved. There were three models with CRP values of 100%, which were the SPA-LS-SVM, UVE-LS-SVM, and CARS-LS-SVM models. Since the SPA-LS-SVM model had the smallest number of input variables, which was 7, it was considered that the SPA-LS-SVM model was the best.

### 3.5. Classification by All Sample Data

To evaluate the overall performance of the hyperspectral imaging technique, classification models for identifying the five classes of samples (leaf vein, healthy mesophyll, slight damage, serious damage, and *Diaphania pyloalis* larva) were established by the PLS-DA, LS-SVM, and LDA algorithms, respectively. The matrixes of both the visible range data and NIR range data were used to establish the models, and the results are shown in Table 4.

Analyzing the results of the visible range data, the F-LS-SVM model was slightly better than the F-PLS-DA model (the CRP values were 91.89% and 86.49%, respectively). After the calculation of the variable selection, the CRP values of the other three LS-SVM models were all higher than 91%, except for the SPA model based on the selected variables. The SPA calculation selected only six variables, but its CRP value was low. The CRP value of the UVE and CARS models exceeded 90%. However, their selected variables were 229 and 34, respectively. The best model was the UVE-SPA-LS-SVM model, which had the CRP of 97.30% and only nine input variables.

For the NIR range data, the results of the F-LS-SVM were also better than that of the F-PLS-DA model. The CRP value was 92.31% for the F-LS-SVM model, and 84.62% for the F-PLS-DA model. Then, the variable selection was performed. The best model was the CARS-LS-SVM model, whose CRP value was 97.44% and the number of selected variables was 21. The results of the SPA-LS-SVM model and the UVE-LS-SVM model were similar to those of the full variables, but the number of variables of the UVE-LS-SVM model was much larger than that of the SPA-LS-SVM model (137 vs. 10). After the SPA calculation was carried out based on the variables selected by the UVE, the number of input variables was reduced to 14, and the established UVE-SPA-LS-SVM models had the results similar to that of the F-LS-SVM model. The above results showed that both the visible range data and NIR range data could identify damage and *Diaphania pyloalis* larvae from the leaves.

## 4. Discussion

Mulberry leaves are important for the sericulture industry. Mulberry planting management has always been the focus of mulberry farmers. Mulberry leaf pest is an important factor affecting the yield and quality of the mulberry leaves. Nowadays, the mulberry leaves are suffering damage from *Diaphania pyloalis*. *Diaphania pyloalis* is a common insect in mulberry fields. The breakout of them often causes a sharp decrease in the production of mulberry leaves. In this work, a hyperspectral imaging technique in the spectral ranges of the visible and short-wave NIR and long-wave NIR was used for the detection and identification of *Diaphania pyloalis* larvae and their damage to mulberry leaves, in a rapid and objective way. The best models for the classification of leaf vein, healthy mesophyll, slight damage and serious damage (classification I); the classification of leaf vein, healthy mesophyll, and *Diaphania pyloalis* larva (classification II); and the classification of slight damage, serious damage, and *Diaphania pyloalis* larva (classification III) were the SPA-LS-SVM models based on the NIR range data, and all of them obtained the CRP values of 100% and AB_CR values of 0%. When all five classes of ROIs, namely leaf vein, healthy mesophyll, slight damage, serious damage, and *Diaphania pyloalis* larva, were considered for classification (classification IV), the best model was the UVE-SPA-LS-SVM model based on the visible range data, which had the CRP value of 97.30% and AB_CR of only 2.70%. Therefore, it was confirmed the potential of the visible and NIR hyperspectral imaging for the identification of *Diaphania pyloalis* larvae and the damage on mulberry leaves. This rapid and accurate detection technique will help mulberry growers to control insect pests and to provide mulberry leaves of a high quality.

**Figure 2:**
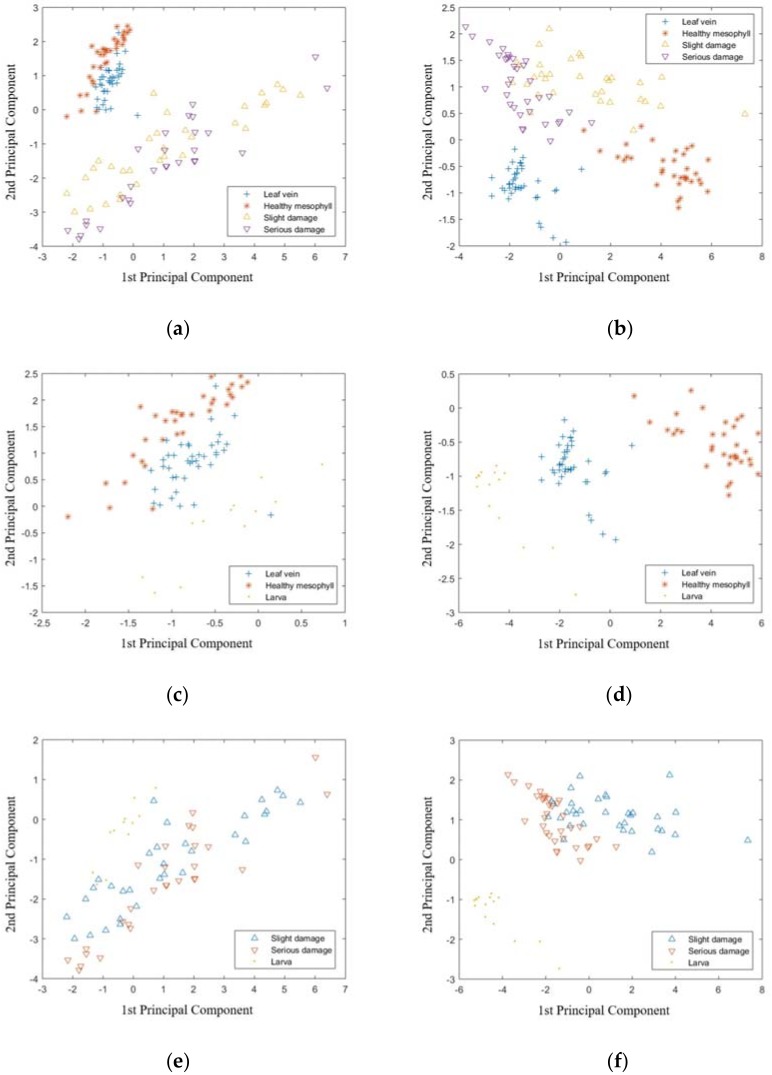

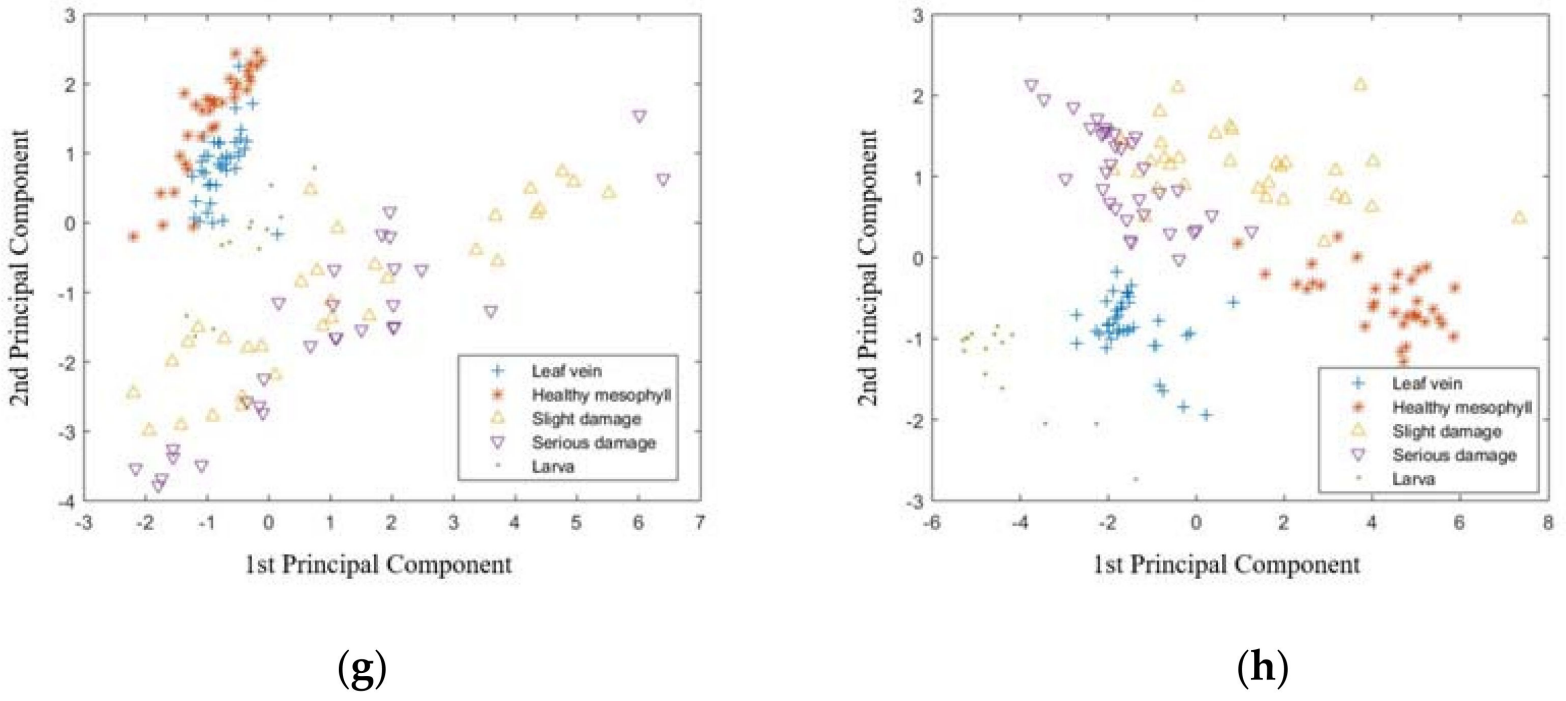
Score plot of the first two principal components of Classification I to IV based on visible range data and NIR range data. Classification I: (**a**) visible range (400–1000 nm) and (**b**) NIR range (900–1700 nm). Classification II: (**c**) visible range (400–1000 nm) and (**d**) NIR range (900–1700 nm). Classification III: (**e**) visible range (400–1000 nm) and (**f**) NIR range (900–1700 nm). Classification IV: (**g**) visible range (400–1000 nm) and (**h**) NIR range (900–1700 nm).

Besides PLS-DA and LS-SVM, the principal component analysis (PCA) and linear discriminant analysis (LDA) were also used to distinguish the samples in classification I to IV, based on the visible range data and NIR range data, respectively. The score plot of the first two principal components is shown in Figure 2. For classification I (Figure 2a,b), samples of leaf vein and healthy mesophyll could be distinguished by the NIR range data and were overlapped based on the visible range data. On the other hand, the samples of slight damage and serious damage were overlapped based on both visible and NIR range data. For classification II (Figure 2c,d), the classification between leaf vein and healthy mesophyll had a similar result to those in classification I. On the other hand, the samples of larvae were mostly clustered, separately. For classification III (Figure 2e,f), samples of the samples with slight damage and serious damage were overlapped based on both the visible and NIR range data. On the other hand, samples of larva were mostly clustered based on the NIR range data, but overlapped with damage samples based on the visible range data. For classification IV (Figure 2g,h), the samples from different classes were overlapped based on the visible range data. On the other hand, on the basis of the NIR range data, the samples of the larva and leaf vein were mostly clustered and those of the healthy mesophyll, slight damage, and serious damage were overlapped. The results of the PCA showed that, compared with the results of LS-SVM, it is not easy to distinguish samples from different classes by analyzing the score plot of the first two principal components. The LDA models were established based on the full variables in the visible and NIR range data for classifications I to IV, and the results are shown in Table 1, Table 2, Table 3 and Table 4. For classifications I and II, the LDA models had a good prediction, based on the NIR range data, but were poorer than the corresponding LS-SVM models. For the Classification III, the LDA models had 100% CRP based on both visible and NIR range data. For classification IV, the results of the LDA models were better than the corresponding PLS-DA models, but poorer than the corresponding LS-SVM models. In general, the results of the LS-SVM models were better than those of the LDA models.

Two spectral matrices of the visible range data and NIR range data were used for the model calibration. It was noted that there were five PLS-DA and five LS-SVM models for both the visible range data and NIR range data in the calculation of classifications I to IV, respectively. The five models for both the PLS-DA and LS-SVM were one full variable model and four models after the variable selection (SPA, UVE, UVE-SPA, and CARS). To evaluate the performances of the visible range data and NIR range data, the average CRP of the five PLS-DA models and the five LS-SVM models were calculated in the calculation of classifications I to IV, respectively. For classification III, both the visible range data and NIR range data had a good prediction. The average CRP values of the five PLS-DA models and the five LS-SVM models were 97.78% and 100.00% for the visible range data and 94.29% and 99.05% for the NIR range data. For classification IV, the visible range data and NIR range data had a similar performance. The average CRP values of the five LS-SVM models of the visible range data and NIR range data were 91.89% and 94.36%, respectively. Nevertheless, those of the five PLS-DA models of the visible range data and NIR range data were only 79.96% and 77.43%, respectively. For classification I, the average CRP values of the five PLS-DA models of the visible range data was only 80.00%, while the average CRP values of the five LS-SVM models of the visible range data and the two average CRP values of the NIR range data were all over 95.00%. For classification II, the average CRP values of the five LS-SVM models of both the visible range data and NIR range data were 95.45% and 100%, respectively, whereas the five PLS-DA models of both the visible range data and the NIR range data did not obtain good results. The above results showed that when LS-SVM was considered, the visible range data and NIR range data were both good at the classification; when PLS-DA was used for model calibration, only the NIR range data for classification I and both the visible range data and NIR range data for classification III obtained a good prediction. Moreover, the above results showed that the LS-SVM algorithm was more suitable than the PLS-DA algorithm for analyzing the hyperspectral image data of the leaves and insect pests. This might be because PLS-DA establishes linear relationships, whereas LS-SVM establishes non-linear relationships.

Variable selection is a common method for improving the accuracy and robustness, as well as reducing the input variables of near-infrared spectroscopy models. In this work, four variable selection methods were employed. In most cases, the accuracy of the model based on the selected variables was similar to that of the model based on the full variables, demonstrating that the variable selection was efficient to remove the irrelevant variables of the spectral data and could maintain or even improve the model accuracy. All the best models for classifications I to IV were based on the variable selection. In specific, SPA was used in the best models for classifications I, II, and III, and UVE-SPA was used in the best model for classification IV. The number of variables selected by CARS is generally dozens, while the number of variables selected by UVE exceeds one hundred. Table 5 shows the selected wavelength variables of the best models for classifications I to IV. These selected variables could maximize the differences among the different classes, thereby establishing classification models that are highly accurate. In general, when the LS-SVM was used to establish the classification models, both the visible and NIR spectra obtained good classification results. The NIR spectrum is the absorption of the overtone and combination vibrations of C–O, C–H, O–H, and other bonds. The selected variables in the NIR region could reflect the spectral difference of the chemical bonds of the samples from different classes. However, because of the serious overtone and the combination of the NIR spectrum, it is difficult to attribute the selected wavelength variables to specific chemical bonds. On the other hand, the visible spectrum is mainly based on the difference of color between different classes of samples in their classification. The green color of leaf vein and healthy mesophyll was mainly because of chlorophyll, whereas the damage caused the decreased presence of chlorophyll, leading to a different absorption of the damage in the red-visible light. Furthermore, the larvae were mainly dark green. It can be said that visible spectra were an important classification basis in this work. However, because of the close color between the samples from different classes or even in the same class, it was difficult to achieve an accurate classification using the machine vision technique, which has only three channel wavelengths, red, green, and blue, in the visible spectrum. There was a need for techniques such as hyperspectral imaging or multispectral imaging that have dozens or several bands in the visible spectrum to perform the classification.

Mulberry leaves are the main food source of silkworms in sericulture. The quality and yield of mulberry leaves are very important. At present, mulberry leaf production is suffering the loss caused by *Diaphania pyloalis* larvae. The established classification models in this study can be used to detect *Diaphania pyloalis* larvae and their damage on mulberry leaves, in order to provide silkworms with high-quality mulberry leaves as feed, and could be beneficial for increasing the yield of silkworms and improving the quality of silk. It should be noted that, currently, the cost of the current hyperspectral imaging system is still high. Therefore, this technique is mainly focused on the determination of optimal wavelengths for the development of multispectral imaging systems with low cost, which will play a significant role in the detection of plant diseases and insect pests. Compared with a hyperspectral imaging system, the cost of a multispectral imaging system is much lower. In the future works, more leaves and larvae samples at different growth stages from different years will be considered. Then, the final optimal wavelengths can be determined by analyzing these samples to further produce a low-cost multispectral imaging system for industrial purpose.

## 5. Conclusions

This study investigated the feasibility of using Vis-NIR hyperspectral imaging to distinguish the *Diaphania pyloalis* larvae and their damage to mulberry leaves in a rapid and nondestructive manner. The classification models were establish based on the extracted spectral data, which was analyzed by multivariable calibration and variable selection methods. The results show that the leaf vein, healthy mesophyll, slight damage, serious damage, and larva could be successfully distinguished from each other (CRP value of 97.30% and AB_CR of only 2.70%). The LS-SVM algorithm was more suitable than the PLS-DA algorithm for model calibration; and in most cases, the variable selection could improve the accuracy of the models. As the first study in rapid identifying *Diaphania pyloalis* larvae and its damage on mulberry leaves using hyperspectral imaging, the results would help provide silkworms with high-quality mulberry leaves. Nevertheless, the practical implementation of a hyperspectral imaging system still faces the shortages of complex calculation and high cost, making it mainly limited to laboratory research. Therefore, it is important to consider more samples to finally determine some key wavelengths to develop an optimized multispectral imaging system for industrial purpose.

## Figures and Tables

**Table 1:** Results of classification models for identifying four classes of samples (leaf vein, healthy mesophyll, slight damage, and serious damage), based on the matrixes of visible range data and near-infrared (NIR) range data. Visible range data is within the spectral range of 400–1000 nm. NIR range data is within the spectral range of 900–1700 nm.

Data	Variable Selection	Variable Number	Calibration	CRC ^a^	CRP ^b^	AB_CR ^c^
visible	/	428	PLS-DA	100.00%	82.35%	17.65%
visible	/	428	LS-SVM	100.00%	97.06%	2.94%
visible	/	428	LDA ^g^	100.00%	88.24%	11.76%
visible	SPA ^d^	10	PLS-DA	98.00%	79.41%	18.59%
visible	SPA ^d^	10	LS-SVM	100.00%	94.12%	5.88%
visible	UVE ^e^	266	PLS-DA	100.00%	79.41%	20.59%
visible	UVE ^e^	266	LS-SVM	100.00%	100.00%	0.00%
visible	UVE-SPA	9	PLS-DA	86.00%	76.47%	9.53%
visible	UVE-SPA	9	LS-SVM	100.00%	94.12%	5.88%
visible	CARS ^f^	28	PLS-DA	100.00%	82.35%	17.65%
visible	CARS ^f^	28	LS-SVM	100.00%	94.12%	5.88%
NIR	/	256	PLS-DA	100.00%	97.22%	2.78%
NIR	/	256	LS-SVM	100.00%	97.22%	2.78%
NIR	/	256	LDA ^g^	100.00%	94.44%	5.56%
NIR	SPA ^d^	9	PLS-DA	97.14%	97.22%	0.08%
NIR	SPA ^d^	9	LS-SVM	100.00%	100.00%	0.00%
NIR	UVE ^e^	180	PLS-DA	100.00%	97.22%	2.78%
NIR	UVE ^e^	180	LS-SVM	100.00%	97.22%	2.78%
NIR	UVE-SPA	9	PLS-DA	97.14%	94.44%	2.70%
NIR	UVE-SPA	9	LS-SVM	100.00%	97.22%	2.78%
NIR	CARS ^f^	20	PLS-DA	100.00%	94.44%	5.56%
NIR	CARS ^f^	20	LS-SVM	100.00%	97.22%	2.78%

^a^ correct rate of calibration, ^b^ correct rate of prediction, ^c^ the absolute difference between the correct rate of calibration and prediction, ^d^ successive projections algorithm, ^e^ uninformation variable elimination, ^f^ competitive adaptive reweighted sampling, ^g^ linear discriminant analysis.

**Table 2:** Results of classification models for identifying three classes of samples (leaf vein, healthy mesophyll, and *Diaphania pyloalis* larva) based on the matrixes of the visible range data and NIR range data. Visible range data is within the spectral range of 400–1000 nm. NIR range data is within the spectral range of 900–1700 nm.

Data	Variable Selection	Variable Number	Calibration	CRC	CRP	AB_CR
visible	/	428	PLS-DA	100.00%	90.91%	9.09%
visible	/	428	LS-SVM	100.00%	95.45%	4.55%
visible	/	428	LDA	100.00%	81.82%	18.18%
visible	SPA	6	PLS-DA	98.44%	77.27%	21.17%
visible	SPA	6	LS-SVM	100.00%	95.45%	4.55%
visible	UVE	108	PLS-DA	89.06%	81.82%	7.24%
visible	UVE	108	LS-SVM	100.00%	95.45%	4.55%
visible	UVE-SPA	5	PLS-DA	85.94%	86.36%	0.42%
visible	UVE-SPA	5	LS-SVM	100.00%	95.45%	4.55%
visible	CARS	43	PLS-DA	100.00%	86.36%	13.64%
visible	CARS	43	LS-SVM	100.00%	95.45%	4.55%
NIR	/	256	PLS-DA	92.42%	60.87%	31.55%
NIR	/	256	LS-SVM	100.00%	100.00%	0.00%
NIR	II	256	LDA	100.00%	95.65%	4.35%
NIR	SPA	9	PLS-DA	63.64%	60.87%	2.77%
NIR	SPA	9	LS-SVM	100.00%	100.00%	0.00%
NIR	UVE	156	PLS-DA	77.27%	65.22%	12.05%
NIR	UVE	156	LS-SVM	100.00%	100.00%	0.00%
NIR	UVE-SPA	15	PLS-DA	65.15%	60.87%	4.28%
NIR	UVE-SPA	15	LS-SVM	100.00%	100.00%	0.00%
NIR	CARS	13	PLS-DA	95.45%	60.87%	34.58%
NIR	CARS	13	LS-SVM	100.00%	100.00%	0.00%

**Table 3:** Results of classification models for identifying three classes of samples (slight damage, serious damage, and *Diaphania pyloalis* larva) based on the matrixes of the visible range data and NIR range data. Visible range data is within the spectral range of 400–1000 nm. NIR range data is within the spectral range of 900–1700 nm.

Data	Variable Selection	Variable Number	Calibration	CRC	CRP	AB_CR
visible	/	428	PLS-DA	100.00%	100.00%	0.00%
visible	/	428	LS-SVM	100.00%	100.00%	0.00%
visible	/	428	LDA	100.00%	100.00%	0.00%
visible	SPA	13	PLS-DA	100.00%	100.00%	0.00%
visible	SPA	13	LS-SVM	100.00%	100.00%	0.00%
visible	UVE	249	PLS-DA	100.00%	100.00%	0.00%
visible	UVE	249	LS-SVM	100.00%	100.00%	0.00%
visible	UVE-SPA	16	PLS-DA	98.15%	88.89%	9.26%
visible	UVE-SPA	16	LS-SVM	100.00%	100.00%	0.00%
visible	CARS	20	PLS-DA	100.00%	100.00%	0.00%
visible	CARS	20	LS-SVM	100.00%	100.00%	0.00%
NIR	/	256	PLS-DA	100.00%	95.24%	4.76%
NIR	/	256	LS-SVM	100.00%	100.00%	0.00%
NIR	/	256	LDA	100.00%	100.00%	0.00%
NIR	SPA	7	PLS-DA	100.00%	90.48%	9.52%
NIR	SPA	7	LS-SVM	100.00%	100.00%	0.00%
NIR	UVE	106	PLS-DA	98.36%	95.24%	3.12%
NIR	UVE	106	LS-SVM	100.00%	100.00%	0.00%
NIR	UVE-SPA	8	PLS-DA	98.36%	95.24%	3.12%
NIR	UVE-SPA	8	LS-SVM	100.00%	95.24%	4.76%
NIR	CARS	26	PLS-DA	100.00%	95.24%	4.76%
NIR	CARS	26	LS-SVM	100.00%	100.00%	0.00%

**Table 4:** Results of classification models for identifying five classes of samples (leaf vein, healthy mesophyll, slight damage, serious damage, and *Diaphania pyloalis* larva) based on the matrixes of the visible range data and the NIR range data. Visible range data is within the spectral range of 400–1000 nm. NIR range data is within the spectral range of 900–1700 nm.

Data	Variable Selection	Variable Number	Calibration	CRC	CRP	AB_CR
visible	/	428	PLS-DA	98.17%	86.49%	11.68%
visible	/	428	LS-SVM	100.00%	91.89%	8.11%
visible	/	428	LDA	100.00%	89.19%	10.81%
visible	SPA	6	PLS-DA	77.06%	86.28%	9.22%
visible	SPA	6	LS-SVM	100.00%	86.49%	13.51%
visible	UVE	229	PLS-DA	98.17%	81.08%	17.09%
visible	UVE	229	LS-SVM	100.00%	91.89%	8.11%
visible	UVE-SPA	9	PLS-DA	71.56%	59.46%	12.10%
visible	UVE-SPA	9	LS-SVM	100.00%	97.30%	2.70%
visible	CARS	34	PLS-DA	100.00%	86.49%	13.51%
visible	CARS	34	LS-SVM	100.00%	91.89%	8.11%
NIR	/	256	PLS-DA	99.15%	84.62%	14.53%
NIR	/	256	LS-SVM	99.15%	92.31%	6.84%
NIR	/	256	LDA	100.00%	89.74%	10.26%
NIR	SPA	10	PLS-DA	72.65%	71.79%	0.86%
NIR	SPA	10	LS-SVM	99.15%	94.87%	4.28%
NIR	UVE	137	PLS-DA	82.05%	71.79%	10.26%
NIR	UVE	137	LS-SVM	99.15%	94.87%	4.28%
NIR	UVE-SPA	14	PLS-DA	74.36%	69.23%	5.13%
NIR	UVE-SPA	14	LS-SVM	100.00%	92.31%	7.69%
NIR	CARS	21	PLS-DA	98.29%	89.74%	8.55%
NIR	CARS	21	LS-SVM	99.15%	97.44%	1.71%

**Table 5:** Optimal wavelengths selected by variable selection methods of the best models for the classifications I to IV.

Classification	Number	Optimal Wavelengths (nm)
I	9	1014, 1393, 1582, 1652, 1655, 1695, 1698, 1705, 1712
II	9	891, 917, 1347, 1453, 1652, 1675, 1695, 1722, 1725
III	7	962, 1085, 1406, 1588, 1685, 1702, 1718
IV	9	393, 410, 430, 450, 560, 640, 677, 687, 703
